# Genome Sequence of Human Herpesvirus 7 Strain UCL-1

**DOI:** 10.1128/genomeA.00830-13

**Published:** 2013-10-10

**Authors:** Callum D. Donaldson, Duncan A. Clark, I. Michael Kidd, Judith Breuer, Daniel D. Depledge

**Affiliations:** MRC/UCL Centre for Medical Molecular Virology, Division of Infection and Immunity, University College London, London, United Kingdoma; Department of Virology, Barts Health NHS Trust, London, United Kingdomb; Department of Virology, University College London Hospitals NHS Foundation Trust, London, United Kingdomc

## Abstract

The sequence of human herpesvirus 7 (HHV-7) strain UCL-1 was determined using target enrichment and next-generation sequencing methods. We have identified 86 putative open reading frames (ORFs), and comparative sequence analyses demonstrate that this strain is closely related to the previously sequenced HHV-7 strains RK and JI.

## GENOME ANNOUNCEMENT

Together with the human herpesviruses (HHVs) HHV-6A and HHV-6B, HHV-7 makes up the *Roseolovirus* genus of the *Betaherpesvirinae* subfamily. Similarly to the other roseoloviruses, HHV-7 has a characteristic genome structure consisting of a central unique long region (U_L_) flanked on either side by direct repeats (DR_L_ and DR_R_) (1). HHV-7 infection can sometimes cause exanthem subitum and has been associated with a number of other diseases, including encephalopathy (2, 3). The majority of children are seropositive to HHV-7 virus by the age of 6 (4), but despite its ubiquity and association with disease, there is a relative paucity of genetic information and understanding.

The first complete HHV-7 genome (strain JI) was published in 1996 (5) using virus DNA isolated from the peripheral blood mononuclear cells of a chronic fatigue syndrome patient (6). This was followed by the publication of HHV-7 (strain RK), isolated from the blood of a healthy donor in 1998 by Megaw et al. (7). Comparative sequence analyses showed these strains to be highly conserved, with particular variation found in the telomere-like repeat regions (7).

Here, for the first time since 1998, we report the sequence of an HHV-7 isolate, strain UCL-1. This virus was originally isolated in 1994 from the saliva of a healthy adult. It was initially cultured in cord blood mononuclear cells before passage in Sup-T1 cells. Total DNA was extracted, sheared, and an Illumina sequence library was prepared prior to target enrichment by hybridization with RNA oligonucleotide baits (the methodology is outlined in Depledge et al. [8]). Paired-end sequencing was then performed using an Illumina MiSeq followed by *de novo* genome assembly using the CLC Genomics Workbench 6. PCR and Sanger sequencing were used to confirm selected single nucleotide polymorphisms (SNPs), insertions, and deletions, and also to determine the sequence of the R1 repeat region. The R2 repeat region could not be solved by conventional methods and is represented as 1,600 bp of ambiguities (Ns).

The genome of strain UCL-1 is 151,471 bp (excluding the R2 region), with the DRs and U_L_ being 10,031 bp and 131,059 bp, respectively. Comparative analyses against HHV-7 (strain RK) revealed identical numbers and sizes of putative open reading frames (ORFs) (86), while phylogenetic analyses showed that this virus strain is closely related to HHV-7 strains JI and RK. Comparative analyses with the existing HHV-7 genomes (strains JI and RK) found few SNP differences within ORFs, with no clearly discernible distribution pattern across the genome. The sequence identities with strains JI and RK were 99.96% and 99.83%, respectively. This high level of conservation may reflect the recent divergence of HHV-7 and/or a highly evolved virus.

### Nucleotide sequence accession number.

The genome sequence of HHV-7 strain UCL-1 can be found in GenBank accession no. KF558370.
